# Identification of Stress Location During Low-Speed Mobility Travel Using Environmental Data

**DOI:** 10.3390/s26061859

**Published:** 2026-03-15

**Authors:** Narumon Jadram, Yuri Nishikawa, Midori Sugaya

**Affiliations:** 1 Shibaura Institute of Technology, Graduate School of Engineering and Science, Functional Control Systems, 3-7-5 Toyosu, Koto-ku, Tokyo 135-8548, Japan; doly@shibaura-it.ac.jp; 2 National Institute of Advanced Industrial Science and Technology, 2-3-26 Aomi, Koto-ku, Tokyo 135-0064, Japan; nishikawa.yuri@aist.go.jp

**Keywords:** low-speed mobility devices, electric wheelchair, stress location, heart rate variability

## Abstract

This study proposes an exploratory framework for identifying stress locations during travel with low-speed mobility devices (LMDs), such as electric wheelchairs. In this framework, stress factors perceived during LMD travel were identified through a post-ride questionnaire, and the travel route was divided into 100 m segments to enable location-specific stress evaluation. The identified factors were quantified using environmental data to construct an environment-based stress estimation index. Based on these quantified factors, a Composite Stress Score (CSS) was calculated to estimate stress levels along the route. Experiments with healthy adult participants were conducted to examine the feasibility of the proposed method. The results identified poor road surface conditions and vibrations, encounters with other road users, and narrow sidewalks as key stress factors during LMD travel. To examine whether the proposed method captures stress-related responses, correlations between CSS-based stress estimates and heart rate variability (HRV) indices were analyzed. The results showed that CSS calculated from poor road surface/vibrations, encounters with other road users, and narrow sidewalks exhibited moderate negative correlations with SDNN, suggesting that higher CSS values may correspond to increased physiological stress responses. These findings provide preliminary support for the exploratory feasibility of estimating potential stress locations during LMD travel using environmental data. However, the generalizability of the results is limited due to the specific experimental route and the use of healthy adult participants.

## 1. Introduction

### 1.1. Background

In Japan, the use of low-speed mobility devices (LMDs), such as electric wheelchairs and mobility scooters, has gained attention as a measure to ensure transportation options for older adults and individuals with mobility difficulties. LMDs are defined as single-occupant electric vehicles that operate at low speeds. Under the Japanese Road Traffic Act, LMDs with a maximum speed of 6 km/h or less are treated as “pedestrians,” allowing their operation on sidewalks without the need for a driver’s license [1]. LMDs are relatively easy to operate and allow users to travel with a low physical burden. As an effective means of supporting short-distance travel, the number of LMD users, including older adults, has been increased in recent years [2].

Despite their practicality, the outdoor use of LMDs may impose psychological burdens (i.e., stress) on users due to various environmental factors, such as traffic conditions and sidewalk characteristics. For example, Torkia et al. reported that uneven surfaces, steps, curbs, and other irregularities on sidewalks, as well as navigating through crowds, make operating electric wheelchairs difficult [3]. Stress caused by such factors can reduce travel comfort and may hinder the continued use of LMDs [4]. One effective approach to mitigating travel-related stress is to identify and avoid locations where stress is likely to occur (hereafter referred to as stress locations). When multiple routes to a destination are available, providing information on stress locations can allow users to compare alternative routes and select those associated with lower levels of stress. Even when only a single route to a destination is available, identifying stress locations enables users to anticipate potential difficulties and prepare appropriate coping strategies in advance. Therefore, the development of methods for identifying stress locations is essential for supporting safe and comfortable mobility using LMDs.

### 1.2. Research on Identifying Stress Locations

To date, several studies have investigated the identification of stress locations during outdoor mobility, such as walking or cycling [5,6,7,8]. These studies primarily employ physiological-based methods to evaluate stress. Physiological-based methods objectively and continuously evaluate stress by analyzing physiological signals such as heart rate (HR), heart rate variability (HRV), electrodermal activity (EDA), and skin temperature (ST) [9]. By associating stress evaluated using physiological indices with location information obtained from global positioning systems (GPS), stress locations can be identified. For example, Kyriakou et al. [5] proposed a stress detection method based on EDA and ST acquired using wearable sensors and applied spatial hotspot analysis. Their results demonstrated that the identified stress locations showed a high correlation with subjective evaluations and observations derived from video recordings. Kim et al. [6] investigated the use of EDA, HR, and gait patterns to capture environmental stress experienced by pedestrians in real-life settings. Using spatial analysis and machine learning models, they demonstrated that location-based collective pedestrian stress could be predicted with an accuracy of approximately 80%. LaJeunesse et al. [7] examined pedestrian stress using EDA, HR, and GPS data recorded over all walking trips during a one-week period, and found that pedestrian stress was more strongly associated with surrounding roadway characteristics and land-use contexts than with specific crossing locations. Furthermore, Dash et al. [8] presented a method for measuring pedestrian stress using HRV to identify high-stress locations. Their results indicated that elevated stress was associated with pedestrian–scooter interactions, high foot-traffic areas, and poor visibility at crossings. These studies demonstrate the potential of physiological-based methods for identifying stress locations. However, several limitations arise when applying such methods. First, movement during outdoor mobility are likely to introduce motion artifacts into physiological signals, which may degrade the accuracy and reliability of stress evaluation [9,10]. Second, reliable physiological measurement typically requires continuous wear with stable skin contact, which imposes significant physical and psychological burdens on users [11,12]. Consequently, measuring physiological signals in real-world environments and over long-term monitoring periods remains challenging. Due to these limitations, identifying stress locations solely based solely on physiological indicators remain challenging.

To address these challenges, stress estimation approaches based on environmental data have been proposed [13,14,15,16]. These approaches estimate an individual’s stress level through the quantitative assessment of stress-related factors. For example, Li et al. [13] proposed a deep learning system, CyclistAI, that estimates cycling stress using acceleration data and noise levels obtained from smartphone sensors. Their evaluation under various environmental conditions demonstrated that CyclistAI achieved a stress-level classification accuracy exceeding 84%. In addition, Mekuria et al. [15] developed the Level of Traffic Stress (LTS) scale to quantify cyclists’ stress levels based on roadway characteristics such as traffic speed, traffic volume, and the number of lanes. By identifying road segments where cyclists are likely to experience high stress levels, the LTS scale enables the evaluation of cycling infrastructure and the prioritization of infrastructure improvements [17]. Building on this concept, Lin et al. [16] proposed AutoLTS, a deep learning framework that automatically evaluates cycling stress based on LTS using street-view images. AutoLTS estimates the LTS level of each road segment from visual features of the road environment and enables city-scale stress assessment by leveraging publicly available imagery, such as Google Street View. These findings suggest that environment-based approaches offer an effective means of complementing physiological-based methods by mitigating motion-induced accuracy degradation and facilitating large-scale identification of stress locations during outdoor mobility.

### 1.3. Issues

Because environment-based stress estimation methods infer stress from quantitative assessments of stress-related factors rather than directly measuring users’ perceived stress, the estimated stress may deviate from the stress actually experienced by users. In particular, using factors that are weakly associated with stress can degrade estimation accuracy. Therefore, accurate environment-based stress estimation requires the appropriate selection and quantitative assessment of stress-related factors. To date, most studies on environment-based stress estimation and stress location identification have focused on walking and cycling [13,14,15,16]. These methods are designed based on stress-related factors assumed to be relevant to these modes of transportation. However, stress-related factors vary depending on the mode of transportation. Previous studies have reported that factors identified as stress factors for one transportation mode may not induce stress in another mode [18]. For example, vibrations transmitted from the road surface constitute a significant stress factor for electric wheelchair users [19], whereas they have a relatively minor impact on pedestrians and are not generally regarded as a stress factor. Furthermore, even under identical environmental conditions, stress responses vary across transportation modes [20,21]. Accordingly, directly applying an environment-based index developed for one mode to another mode may cause a mismatch between the factors used for estimation and those that actually cause stress, potentially resulting in inaccurate stress estimation.

Therefore, for emerging transportation modes such as LMDs, a methodology is needed to construct an environment-based stress estimation index grounded in mode-specific stress factors and relevant environmental data. However, to the best of our knowledge, such a methodology has not yet been established. For LMD travel, stress factors have not been sufficiently identified, and it remains unclear whether an index constructed based on these factors can accurately reflect the stress experienced during travel.

### 1.4. Purpose and Proposal

This study aims to develop a method for identifying stress locations during LMD travel. To achieve this goal, we propose a framework that constructs an environment-based stress estimation index using data related to LMD-specific stress factors to identify stress locations. We hypothesize that an environment-based stress estimation index based on LMD-specific stress factors is associated with physiological stress responses during travel. The proposed method consists of the following steps:

1. Identification of LMD-specific stress factors: Since the stress factors specific to LMD travel remain unclear, questionnaire responses completed by users after actual LMD travel are analyzed to extract subjectively perceived stress factors.
2. Route segmentation: To enable location-specific stress evaluation, the travel route is divided into fixed-length segments.
3. Quantification of Stress Factors using environmental data: For each segment, the identified stress factors are quantitatively evaluated using corresponding environmental data.
4. Integration into a composite index: To capture the combined effects of multiple stress factors acting simultaneously, the quantified stress factors are integrated into a composite index, referred to as the Composite Stress Score (CSS). The CSS is calculated for each segment, and segments are classified into three stress levels—low, medium, and high—thereby enabling the identification of stress locations.

To examine whether the proposed method can capture stress and identify stress locations during LMD travel, we first examine the association between environment-based stress estimation index (CSS) and physiological stress responses derived from HRV indices using correlation analyses. We then conduct an exploratory case analysis on representative route segments with clearly contrasting CSS-based stress levels to qualitatively illustrate how CSS-based classifications correspond to changes in HRV.

In this study, we conducted experiments with healthy adult participants to assess the real-world feasibility of the proposed stress location identification method. Although LMDs are commonly used by older adults and individuals with mobility impairments, healthy participants were recruited at this initial stage to ensure safety while evaluating the proposed method.

The remainder of this paper is organized as follows. Section 2 presents methodology, experimental design and procedures. Section 3 presents the experimental results. Section 4 discusses the findings, and Section 5 concludes the paper.

## 2. Methodology

### 2.1. Framework of the Proposed Method

#### 2.1.1. Identification of LMD-Specific Stress Factors

To identify subjective stress factors during LMD travel, this study analyzes questionnaire responses collected from users after they completed actual LMD trips.

The questionnaire consisted of three types of questions designed to capture different aspects of the stress experience. First, respondents are asked to provide free-form descriptions of specific locations where they experienced discomfort. Example prompts, such as “around bridges,” are included to encourage detailed responses. This question aims to capture the spatial context and locational features associated with stress occurrences. Second, a multiple-choice question is presented to identify the causes of discomfort. Respondents may select multiple stress factors from a list of eight environmental factors considered potential stressors during LMD travel: pedestrians, bicycles, noise, temperature and humidity, wind, unfamiliar routes, road surface conditions/vibrations, and brightness. An open-ended “Other” option is also provided to allow respondents to describe additional factors not included in the list. Third, a free-response item asks respondents to explain why the selected factors caused discomfort, thereby providing supplementary information about the specific circumstances and context of the stress experience.

Stress factors are identified through a structured coding procedure applied to both multiple-choice and free-text survey responses.

First, responses to the multiple-choice question regarding causes of discomfort are aggregated to calculate the frequency of each predefined stress factor category. The predefined categories are pedestrians, bicycles, noise, temperature and humidity, wind, unfamiliar routes, road surface conditions/vibrations, and brightness. Responses provided under the “Other” option are systematically reviewed and compared against the conceptual definitions of the predefined categories. When semantic equivalence is identified, the response is incorporated into the corresponding existing category. Responses representing conceptually distinct constructions that could not be subsumed under any predefined category were treated as candidate new stress factors and evaluated during the coding process.

Second, free-text responses describing locations and reasons for discomfort are analyzed using a structured deductive coding framework based on the same predefined categories.

The analysis of free-text responses followed a structured three-stage procedure:

1. Frequently occurring terms and characteristic expressions are extracted from each response.
2. Extracted expressions are grouped based on semantic similarity and contextual commonality.
3. Each semantic group is compared with the conceptual definitions of the predefined stress factor categories. When conceptual alignment is confirmed, the group is assigned to the corresponding category. A new category is established only when the content cannot be conceptually integrated into any predefined category and can reasonably be interpreted as an independent stress factor.

Category assignment followed these operational criteria:

- A response is assigned to a category when its core meaning corresponds to the conceptual definition of that stress factor.
- Coding is binary (0 = not present, 1 = present), indicating whether a stress factor is mentioned in the participant’s response.
- A single response may be assigned to multiple categories when multiple independent stress factors are described.
- Minor lexical variations do not justify category differentiation.
- Responses containing no relevant descriptive content (e.g., “no answer”) are excluded from analysis.

For example, descriptions such as “large bumps were frightening” and “vibrations made me feel off balance” are classified under *road surface conditions/vibrations*. In contrast, responses such as “felt scared crossing a narrow bridge” or “felt uneasy on narrow paths” do not align with any predefined category and are therefore grouped into a newly established category, *Narrow sidewalks*.

The responses were independently coded by two researchers using the predefined coding framework and operational definitions described above. After the initial coding, the results were compared to assess the level of agreement between the two coders. When discrepancies were identified, the corresponding responses were re-examined and discussed until agreement was reached. Only the stress factor categories agreed upon by both coders were included in the final analysis, ensuring the reliability of the coding process.

After coding is finalized, the frequency of each stress factor category, both predefined and newly established, is calculated by summing the number of participants for whom the factor is present. This procedure enables the systematic identification of stress factors encountered during LMD travel and the quantification of their frequency across user reports.

#### 2.1.2. Route Segmentation

**Figure 1:**
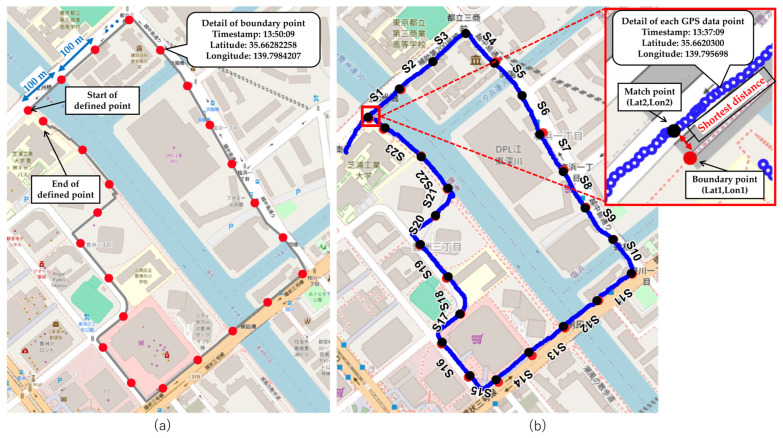
(**a**) Boundary points (red dots) are placed at 100 m intervals along the route, and the segments between adjacent points are used as the analysis units. (**b**) Illustration of the GPS trajectory (blue line) and boundary points used to determine segment entry and exit times. The matched point (black dots) represents the GPS data point with the shortest geodesic distance to the boundary point. S denotes a segment, and the number indicates the segment ID.

Route segmentation is performed to associate estimated stress levels with specific locations along the route. The route is segmented using the QChainage plugin in QGIS [22], which generates segment boundary points at fixed intervals, thereby creating equal-length segments. Each boundary point is assigned a timestamp as well as latitude and longitude information. As illustrated in Figure 1a, boundary points (red dots) are sequentially placed along the route, and the area between two adjacent boundary points is treated as a single analysis segment.

In this study, the segment length is set to 100 m to enable the calculation and comparison of HRV indices for each segment during method validation. Previous studies have reported that at least 60 s of HRV data are required to ensure the reliability of HRV indices [23,24]. Assuming a maximum LMD speed of 6 km/h, a segment length of 100 m ensures that more than 60 s of HRV data can be obtained within each segment. Therefore, a segment length of 100 m was adopted for stress location identification.

#### 2.1.3. Quantification of Stress Factors Using Environmental Data

To quantify stress factors during LMD travel, environmental data corresponding to potential stress factors are collected. These data include the presence of pedestrians and bicycles, noise levels, temperature and humidity, wind conditions, road surface conditions and vibrations, as well as sidewalk-related information. Details of the data acquisition methods are described in Section 2.5.

In this study, the stress factors used for stress estimation were selected based on the distribution of reporting rates observed in the survey results. To improve general applicability, only stress factors commonly reported by a substantial proportion of participants were retained, while factors that may reflect individual-specific conditions were excluded. A natural discontinuity in the distribution of reporting rates was used as the criterion for factor selection.

Stress factors are quantified on a segment-by-segment basis by synchronizing environmental data with GPS data using a common timestamp. The quantification procedure consists of three steps: (1) temporal synchronization of environmental and GPS data, (2) determination of entry and exit times for each segment, and (3) extraction and aggregation of environmental data within the corresponding time interval to compute representative values for each segment.

The entry and exit times of each segment are determined by matching the segment boundary points defined through route segmentation with the GPS trajectory. As illustrated in Figure 1b, geodesic distances are calculated between all GPS observation points and all segment boundary points. For each boundary point, the GPS observation point with the minimum distance is identified and defined as the match point. The timestamp associated with the match point is regarded as the time at which the boundary point is passed and is used as the entry or exit time of the corresponding segment.

The quantified values of each stress factor for each segment are classified into three levels (Levels 1–3) to represent stress factor intensity. For stress factors with established evaluation criteria or predefined threshold values, level classification is performed based on those criteria. For factors without widely accepted thresholds, a quantile-based classification method is applied [25]. This method is robust to differences in data distribution and less sensitive to outliers, enabling segment-level data to be divided into three levels based on relative magnitude. As a result, stress factor intensities can be consistently compared across segments, even when absolute scales differ among stress factors.

#### 2.1.4. Calculation of Composite Stress Score

To integrate the effects of multiple stress factors, the CSS is calculated for each segment based on the classified scores of the quantified stress factors. The CSS for segment i, denoted as ${CSS}_{i}$, is defined as the sum of the classification scores of all stress factors, as given in (1).

(1) $${CSS}_{i}=\sum_{j=1}^{n}S_{i,j}$$

where $S_{i,j}$ denotes the classification score of stress factor j in segment i, and n is the number of stress factors. Since each classification score ranges from 1 to 3, the ${CSS}_{i}$ take values between 1 and 3n. Based on the ${CSS}_{i}$, each segment is classified into three stress levels: Low, Moderate, and High. The ${CSS}_{i}$ range (1–3n) is equally divided into three intervals, and the stress level of segment i is defined as (2)

(2) $${Stress\;Level}_{i}=\{\begin{array}{c} Low,\;\;{CSS}_{i}\leq n\\ Moderate,\;\;{n\leq CSS}_{i}\leq 2n\\ High,\;\;{2n<CSS}_{i}\leq 3n \end{array}$$

For example, when the composite score is calculated based on two stress factors (n=2), segments with a score of 2 or less are classified as Low, those with scores of 3–4 as Moderate, and those with scores of 5–6 as High. By integrating the effects of multiple stress factors into a single composite score, this method enables quantitative evaluation of segment-level stress intensity along the travel route.

### 2.2. Experimental Setup

To evaluate the proposed framework under real-world conditions, field experiments were conducted during actual LMD travel in an urban environment. An electric wheelchair was adopted as a representative example of a low-speed mobility device (LMD).

Experiments were conducted between July and December and were scheduled within four daytime time slots (9:00–10:30, 10:45–12:15, 13:30–15:00, and 15:15–16:45). Data collection was postponed during rainy or otherwise adverse weather conditions to ensure participant safety and data quality. During each trial, an experimenter followed the participant at a safe distance to ensure safety and provide assistance when necessary. The experimenter maintained a non-interfering position and avoided verbal interaction unless safety intervention was required. This experiment was approved by the Ethics Committee of Shibaura Institute of Technology (Approval No. 23-048).

### 2.3. Participants

A total of 37 participants (age range: 19–32 years; mean age: 23.3 ± 3.8 years; 15 females) provided informed consent and participated in the experiment. The participants were from nine different countries. Participants were recruited within the university and received compensation of 1800 JPY for their participation.

Healthy adult participants were recruited to ensure the safety of the experimental procedures before extending the study to target populations such as older adults or individuals with mobility difficulties. Because the experiment involved outdoor travel using an electric wheelchair, all participants received training on the basic operation of the device prior to the experiment. Only participants who were judged capable of safely operating the wheelchair were allowed to participate. All participants completed the experiment following the same experimental protocol and travel route.

Due to technical issues, environmental and video data from three participants could not be recorded; therefore, these participants were excluded from analyses requiring environmental and/or video data. Two additional participants were excluded due to insufficient physiological data quality (see Section 2.7.3). As a result, analyses were conducted with 32 participants.

### 2.4. Experimental Route

**Figure 2:**
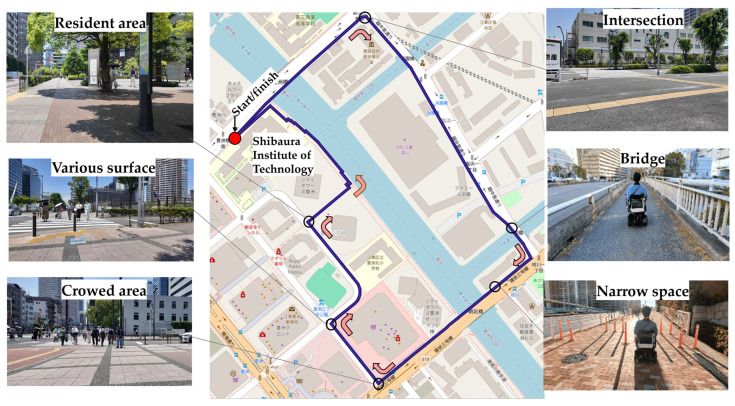
Overview of the experimental route and representative photographs of key environmental contexts along the route (blue line). Participants completed a clockwise loop starting from the Toyosu Campus of the Shibaura Institute of Technology (red dot; start/finish point). White circles indicate the locations of the representative photographs, and curved arrows indicate the direction of travel. The route included residential areas, various pavement surfaces, crowded areas, intersections, a bridge, and narrow spaces. Participants encountered intersections; however, for safety reasons, they were not required to cross any roads.

The experimental route is illustrated in Figure 2. The route was established along sidewalks in the Toyosu area of Koto Ward, Tokyo, Japan, forming a clockwise loop of approximately 2.7 km starting from the Toyosu Campus of Shibaura Institute of Technology. The route was designed to incorporate diverse urban sidewalk environments that LMD users may encounter, including residential streets, bridge sections, narrow sidewalk segments, varying pavement conditions, and areas with relatively high pedestrian density. This diversity enabled evaluation of environmental stress under heterogeneous urban contexts. Although the route included intersections, participants were not required to cross any roadways (e.g., via crosswalks) to reduce traffic-related risks. Intersections were included only as spatial contexts encountered along the sidewalk.

### 2.5. Data Acquisition

#### 2.5.1. Equipment and Sensor Configuration

In this study, an electric wheelchair (WHILL Model CR, WHILL Inc., Tokyo, Japan) was adopted as a representative example of LMD. The wheelchair has a maximum speed of 6 km/h, a cruising range of 18 km, and a maximum climbing capability of 10°. Speed and steering were controlled via a joystick, and an automatic speed-limiting function was activated on steep slopes to ensure operational safety.

**Figure 3:**
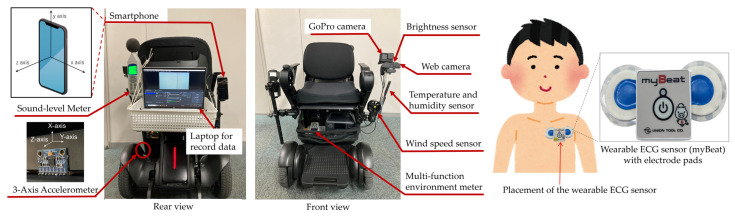
Sensor configuration on the electric wheelchair (WHILL Model CR) and placement of the wearable ECG sensor (myBeat). The wheelchair was equipped with a sound-level meter, three-axis accelerometer, cameras (GoPro and web camera), brightness, temperature and humidity, wind speed sensors, and a multi-function environmental meter. HRV was measured using a wearable ECG sensor attached to the participant’s chest and recorded on a laptop mounted on the wheelchair.

Multimodal sensors were mounted on the wheelchair to enable synchronized data acquisition throughout the trip (Figure 3). The sensor configuration included a sound-level meter, a multifunction environmental meter (measuring temperature, humidity, wind speed, and illuminance), a smartphone for tri-axial acceleration and GPS recording, and cameras for forward-facing video capture. The smartphone used for acceleration and GPS recording was mounted on the electric wheelchair with a standardized orientation. The device was fixed such that the y-axis was aligned vertically upward, and the back camera faced the traveling direction (Figure 3). Physiological signals were recorded using a wearable ECG sensor which was attached to the participant’s chest.

#### 2.5.2. Environmental Data

Noise levels were recorded at 0.5 Hz using a sound level meter (SL-42023SD, Sato Sho-Ji Corp., Japan), with a measurement range of 30–130 dB. Temperature, humidity, wind speed, and illuminance were measured at 0.5 Hz using a multifunction environmental meter (EMC-9400SD, Sato Sho-Ji Corp., Tokyo, Japan). The measurement ranges were 0–50 °C for temperature, 0–95% RH for humidity, 0.4–25.0 m/s for wind speed, and 0–20,000 lux for illuminance. All sensors were connected via cables to the main unit, and the recorded data were stored on an SD card.

To evaluate vibrations during LMD travel, three-axis acceleration data were recorded at 100 Hz using a smartphone rigidly mounted on the electric wheelchair. Although auxiliary acceleration measurements were also obtained using an Arduino microcontroller with a GY-521 (MPU-6050) sensor at 2 Hz, only the smartphone-based acceleration data were used for vibration analysis due to their higher sampling frequency. The orientation of the acceleration axes is shown in Figure 3.

To capture road surface conditions and changes in the surrounding environment, forward-facing video was recorded using a GoPro Hero11 Black. Timestamped video was simultaneously recorded using Open Broadcaster Software (OBS) to enable synchronization with other sensor data. In addition, spatial information related to sidewalks was collected using Google Maps and OpenStreetMap (OSM), including sidewalk width, road classification, and surface type.

#### 2.5.3. GPS Data

GPS data were collected at 1 Hz using the smartphone’s built-in GPS via the Sensor Logger application [26]. Stable GPS reception of latitude and longitude signals was also verified prior to departure. The recorded data included latitude, longitude, altitude, speed, and positioning accuracy.

#### 2.5.4. Physiological Data

Heart rate variability (HRV) represents fluctuations in consecutive interbeat intervals (R–R intervals, RRI) and is a physiological indicator reflecting the activity of the autonomic nervous system (ANS). In this study, HRV was used to evaluate physiological stress during LMD travel.

HRV can be measured using electrocardiography (ECG) or photoplethysmography (PPG); however, PPG is susceptible to motion-induced noise, and its signal quality has been reported to degrade during movement, particularly when using mobility devices [27]. Therefore, ECG was adopted in this study to ensure signal stability. In this study, HRV was measured using a wearable ECG sensor (myBeat WHS-1, Union Tool Co., Tokyo, Japan) [28], which can acquire ECG signals with a 1000 Hz sampling rate, three-axis acceleration, and body surface temperature. As shown in Figure 3, the sensor was attached to the participant’s chest using disposable electrode pads. The acquired signals were processed using standard software for the heart rate sensor (Acc Analyzer, Union Tool Co., Tokyo, Japan) and stored as time-series data of RRI.

#### 2.5.5. Survey

In the experiment, three questionnaire surveys were conducted. The pre-experiment questionnaire collected demographic information and self-reported health conditions. After a rest period, participants completed a questionnaire using the Self-Assessment Manikin (SAM) scale [29] to assess perceived comfort and arousal. In addition, the post-experiment questionnaire included the SAM scale as well as items evaluating riding comfort, perceived discomfort factors (e.g., vibrations and slopes), and qualitative feedback on specific locations where stress or pleasant experiences were encountered during travel. To support participants’ recall of the entire trip during the post-experiment questionnaire, a printed map of the experimental route was provided, and participants were asked to refer to it while answering the survey.

#### 2.5.6. Sensor Calibration

Before each experimental session, all environmental sensors were inspected under stable indoor conditions to confirm proper operation within the manufacturer-specified measurement ranges. During calibration, sensor outputs were monitored for at least 60 s to verify the absence of discontinuities or abnormal fluctuations. If unstable readings were detected, the sensor was reinitialized and the calibration procedure was repeated before the experiment began. In addition, the ECG signal from the wearable sensor was also visually inspected to confirm that a stable ECG waveform was obtained. The signal was monitored for approximately 1 min, and if a stable waveform could not be obtained, the position of the ECG sensor and electrode pads was adjusted.

To verify the validity of the acceleration measurements, the smartphone used for recording tri-axial acceleration and GPS data was checked while the wheelchair was stationary. Acceleration readings along the X, Y, and Z axes were examined to confirm that gravitational acceleration (approximately 9.8 m/s^2^) was primarily detected along the vertical axis, while the remaining axes showed values close to 0 m/s^2^. This procedure ensured correct sensor orientation and offset validity.

After each experimental session, the recorded data were reviewed to confirm successful acquisition without missing segments or recording errors. The verified data were securely saved and backed up. Subsequently, the battery status of all devices was checked, and recharging or battery replacement was performed as necessary to ensure stable operation in subsequent sessions.

### 2.6. Experimental Procedure

The experiment was conducted as follows. After receiving an explanation of the study, written informed consent was obtained from each participant. Participants then completed the pre-experiment questionnaire, after which they attached the ECG sensor in a private room and rested for 2 min to record baseline HRV data. Next, participants received instructions on operating the electric wheelchair and were given time to practice. They then navigated the designated route using the electric wheelchair, with freedom to adjust speed and maneuvering. If a participant forgot the route, they were instructed to refer to a provided A4-sized map or consult the experimenter accompanying them from behind. After completing the route, participants completed the post-experiment questionnaire, the ECG sensor was removed, and the experiment concluded.

### 2.7. Data Processing and Feature Extraction

#### 2.7.1. Evironmental Data Processing

To achieve temporal synchronization with the GPS data, the timestamps of the environmental data (temperature, humidity, wind speed, illuminance, and sound pressure level) were converted to Japan Standard Time (JST). The validity of each dataset was then examined, and samples containing anomalous values were excluded from the analysis. Specifically, measurements exceeding the physical limits of each sensor’s measurement range were classified as anomalous and removed.

Vibration was evaluated using acceleration data. Tri-axial acceleration data acquired using a smartphone were preprocessed as follows. First, UNIX timestamps were converted to JST to ensure temporal synchronization with other sensor data. Next, a band-pass filter with a passband of 0.5–45 Hz was applied to remove low-frequency drift and high-frequency noise components. Subsequently, the mean acceleration of each axis (X, Y, and Z) was calculated during periods when the electric wheelchair was stationary and subtracted from the acceleration data recorded during motion to perform offset correction. Using the corrected data, the root mean square (RMS) of the vertical acceleration was calculated at 1 s intervals [30].

Forward-facing video captured by a camera mounted on the electric wheelchair was processed to detect pedestrians and cyclists encountered during LMD travel and to count their numbers. Pedestrian and cyclist detection was performed using a pretrained YOLOv11m model provided by the Ultralytics YOLO library (version 8.3.49) [31], implemented in Python (version 3.11.10) library. YOLOv11m was selected for this study because it provides a balance between high detection accuracy and inference speed suitable for real-time processing compared with earlier YOLO models [32]. When estimating traffic volume, pedestrians and cyclists were not distinguished; instead, they were aggregated into the “person” class defined in the pretrained model. To improve detection reliability and reduce false positives, only detections with a confidence score of 0.80 or higher were considered. This threshold produced counts that closely matched those obtained through manual observation. Furthermore, to prevent duplicate counting of the same individual and to accurately quantify encounter events, each detected person was assigned a unique ID and tracked across frames. This approach enabled accurate counting of the number of unique individuals encountered at least once during travel. Since this study focused solely on quantifying the frequency of encounters with pedestrians and cyclists, the distance between the LMD user and detected individuals and their apparent size in the video frames were not considered.

#### 2.7.2. GPS Data Processing

GPS data were preprocessed to ensure temporal consistency and data completeness. First, the timestamps were converted to the local time zone to align with other sensor data. Samples with missing latitude or longitude values were removed, and the remaining data were sorted chronologically. To obtain a uniform temporal resolution and facilitate synchronization with environmental data, the GPS trajectory was resampled at 1 Hz. Short gaps caused by temporary signal loss were interpolated using geodesic interpolation [33], whereas segments with prolonged signal loss were excluded from further analysis.

#### 2.7.3. HRV Data Preprocessing

**Figure 4:**
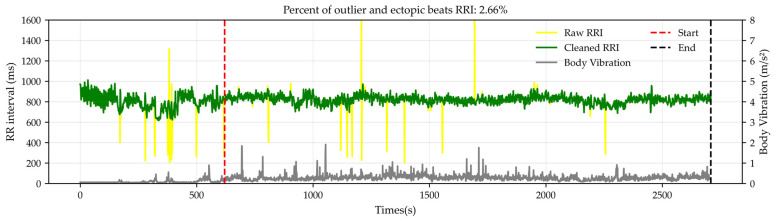
Example of RRI preprocessing. Raw RR intervals (RRI; ms, yellow) and cleaned RRIs after artifact removal (green) are shown together with body vibration (m/s^2^, gray). The red dashed line indicates the start of the LMD riding period, and the black dashed line indicates the end of the analysis segment. In this example, 2.66% of RRIs were identified as outliers or ectopic beats and removed during preprocessing.

During LMD travel, motion artifacts caused by body movement and vibration may contaminate physiological signals. Therefore, prior to HRV analysis, outliers and ectopic beats in the RRI series were removed. First, RR intervals shorter than 300 ms or longer than 1300 ms were treated as ectopic beats and excluded from the analysis [34]. Next, based on previous studies [35], two HRV filtering methods were applied. A Hampel filter was used to identify outliers based on the median absolute deviation (MAD), where the standard deviation was estimated as σ ≈ 1.4826 × MAD. RRIs with deviations exceeding 3σ from the mean were classified as outliers. In addition, a quotient filter was applied to detect abrupt RRI changes; RRIs exhibiting changes greater than 10% compared with the mean of the preceding and following RRIs were also identified as outliers. After filtering, the RRI time series was interpolated using cubic spline interpolation to preserve temporal continuity. Participants for whom more than 5% of the total RRI data were excluded were removed from further analysis due to insufficient data quality. Figure 4 shows an example of the RRI preprocessing results. The raw RRI time series (yellow) exhibits abrupt fluctuations and outliers, which are presumed to be caused by vibration and body movement during LMD operation. In contrast, the preprocessed RRI time series (green) effectively removes these noise components while preserving the overall variability trend. Comparison with the simultaneously recorded body vibration signal (gray) indicates that motion artifacts occur more frequently during periods of higher vibration. In this example, 2.66% of the RRIs were removed, confirming that excessive data rejection was avoided.

#### 2.7.4. HRV Indice Computation

HRV indices are widely used as reliable objective measures for stress evaluation [36,37]. Previous studies have reported that stress induces activation of the sympathetic nervous system (SNS), leading to reduced HRV [38]. HRV analysis can be conducted using time-domain, frequency-domain, and nonlinear methods [39]. Among these, time-domain analysis quantifies the magnitude of HRV fluctuations and has been shown to provide reliable indices even with relatively short recording durations [23,40]. Time-domain HRV indices are also reported to be less sensitive to motion artifacts than frequency-domain indices [41]. Furthermore, the effectiveness of time-domain HRV indices derived from ultra-short-term recordings (30–120 s) has been demonstrated for stress evaluation during walking [42]. Representative time-domain HRV indices include the standard deviation of normal-to-normal intervals (SDNN), the root mean square of successive differences (RMSSD), and the percentage of successive RR interval differences greater than 50 ms (pNN50). SDNN reflects both sympathetic and parasympathetic activity, whereas RMSSD and pNN50 primarily reflect parasympathetic activity [39]. These indices are commonly used to assess stress, with lower values indicating higher stress levels [43]. In this study, SDNN, RMSSD, and pNN50 are adopted as physiological indicators for stress evaluation.

Using the preprocessed RRI data, SDNN, RMSSD, and pNN50 were calculated for each segment according to (3)–(5):

(3) $$\mathrm{SDNN}=\sqrt{\frac{1}{N-1}\sum_{i=1}^{N}{\left({RR}_{i}-\bar{RR}\right)}^{2}}$$

(4) $$\mathrm{RMSSD}=\sqrt{\frac{1}{N-1}\sum_{i=1}^{N}{\left({RR}_{i+1}-{RR}_{i}\right)}^{2}}$$

(5) $$\mathrm{pNN}50=\frac{1}{N-1}\sum_{i=1}^{N}\{\;\left|{RR}_{i+1}-{RR}_{i}\right|>50\;ms\;\}$$

Here, RR denotes the RR interval measured in milliseconds, and $\bar{RR}\;$represents the mean RR interval. After computing these indices, z-score normalization was applied to each HRV metric to account for inter-individual differences in physiological baseline values and to enable comparison across participants.

#### 2.7.5. Data Synchronization

To ensure temporal synchronization across devices, a common reference time was established. Before each experiment, the internal clocks of all sensors were manually aligned with the system time of a notebook computer used for recording HRV data and web camera video. After data collection, the timestamps from all sensors were converted to Japan Standard Time (JST) to ensure consistent temporal alignment. These converted timestamps were then used as a unified temporal reference for integrating environmental, GPS, physiological, and video data streams. Time alignment was verified to the nearest second, resulting in an estimated synchronization error of less than ±1 s across devices.

### 2.8. Statistical Analysis

To examine whether the proposed CSS-based framework can capture stress and identify stress locations during LMD travel, the feasibility of the method was evaluated using HRV indices through the following procedure.

First, to examine whether CSS-based stress estimates are associated with physiological stress responses, Spearman’s rank correlation analysis was conducted between CSS and HRV indices (SDNN, RMSSD, and pNN50). For this analysis, HRV indices were aggregated at the route-segment level by averaging values across participants for each segment; therefore, the effective sample size corresponds to the number of route segments (n = 23). To quantify uncertainty, 95% confidence intervals were estimated using nonparametric bootstrap resampling (5000 iterations). Statistical significance was assessed using two-sided tests, with * *p* < 0.05 and ** *p* < 0.01. With a two-sided significance level of α = 0.05, n = 23 provides approximately 80% power to detect correlations of about |ρ| ≥ 0.56; therefore, smaller associations may not be reliably detected in the present analysis. The interpretation of correlation strength followed the criteria shown in Table 1 [44].

Next, to illustrate how CSS-based classifications correspond to physiological responses, z-score–standardized HRV indices (SDNN, RMSSD, and pNN50) were compared across representative segments with clearly contrasting CSS-based stress levels (high vs. low) using within-subject analyses. Prior to between-segment comparisons, normality was assessed using the Shapiro–Wilk test. When normality was satisfied, a one-way repeated-measures ANOVA was applied; otherwise, the Friedman test was used. For post hoc analysis, paired *t*-tests with Bonferroni correction were conducted when normality was satisfied, whereas the Wilcoxon signed-rank test with Bonferroni correction was used when normality was not satisfied. The significance level was set to α = 0.05.

## 3. Results

### 3.1. Stress Factors Identified by Post-Ride Survey

**Figure 5:**
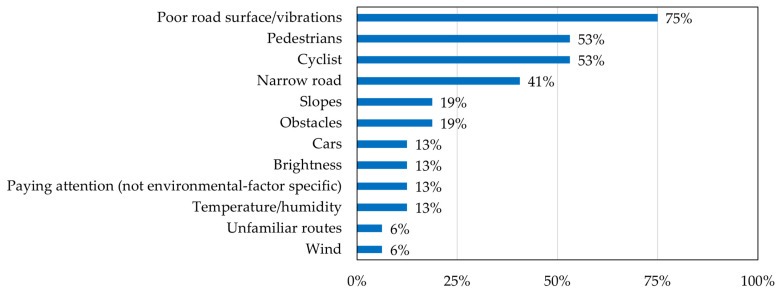
Stress factors identified from the post-ride questionnaire and the percentage of participants reporting each factor (n = 32). Values represent the proportion of participants (%) who identified each item as a stress factor during LMD travel. Participants were allowed to select multiple factors.

Following the procedure described in Section 2.1.3, the results of the post-ride questionnaire were analyzed to identify stress factors perceived during LMD travel. Figure 5 presents the identified stress factors and the percentage of participants who reported each factor. The most frequently reported stress factor was poor road surface/vibrations, reported by 75% of the participants. This was followed by cyclists and pedestrians, each reported by 53% of the participants. Narrow roads were reported by 41% of the participants. Obstacles and slopes were reported by 19% of the participants. Temperature/humidity, paying attention (not environmental-factor specific), brightness, and cars were each reported by 13% of the participants. Wind and unfamiliar routes were each reported by 6% of the participants.

As shown in Figure 5, the top four factors, namely Poor road surface/vibrations (75%), Cyclists (53%), Pedestrians (53%), and Narrow sidewalk (41%), were reported considerably more frequently than the remaining factors (19% or below). A clear gap of more than 20 percentage points exists between the fourth and fifth ranked factors. Based on this natural discontinuity in the distribution, these four factors were selected for CSS design. Because cyclists and pedestrians both result from sharing travel space with other road users, these two factors were integrated into a single factor, referred to as encounters with others. In the subsequent analysis, environmental data corresponding to these stress factors were quantified and used to identify stress locations.

### 3.2. Relationship Between CSS and HRV Indexes

As described in Section 4.1, the stress factors commonly reported by multiple users during LMD travel were poor road surface/vibrations, encounters with others, and narrow sidewalks. To develop an environment-based stress estimation index, each factor was quantified using corresponding environmental measures to calculate the CSS. Specifically, poor road surface/vibrations were represented by the root mean square (RMS) of vertical acceleration, encounters with others were quantified as the number of pedestrians and cyclists detected from onboard video recordings, and narrow sidewalks were represented by sidewalk width.

CSS were calculated for each route segment using either single environmental factors or combinations of multiple factors, following the method described in Section 2.4. The correlation coefficients between CSS and the HRV indices (RMSSD, SDNN, and pNN50) are presented in Table 2.

As shown in Table 2, among the CSS values derived from a single environmental factor, CSS (1), which represents poor road surface/vibrations, showed a moderate negative correlation with SDNN (ρ = −0.487, *p* < 0.05). In contrast, CSS (2), representing encounters with others, and CSS (3), representing sidewalk width, showed no significant correlations with RMSSD, SDNN, or pNN50.

In contrast, CSS values derived from combinations of environmental factors showed associations with multiple HRV indices. CSS (4), which combines poor road surface/vibrations and encounters with others, showed moderate negative correlations with RMSSD (ρ = −0.433, *p* < 0.05) and SDNN (ρ = −0.595, *p* < 0.01). CSS (5), which combines poor road surface/vibrations and sidewalk width, showed a moderate negative correlation with SDNN (ρ = −0.480, *p* < 0.05). CSS (6), which combines encounters with others and sidewalk width, showed moderate negative correlations with RMSSD (ρ = −0.508, *p* < 0.05) and pNN50 (ρ = −0.451, *p* < 0.05).

Furthermore, CSS (7), which integrates all three environmental factors, showed moderate negative correlations with all HRV indices: RMSSD (ρ = −0.525, *p* < 0.05), SDNN (ρ = −0.627, *p* < 0.01), and pNN50 (ρ = −0.449, *p* < 0.05).

### 3.3. Exploratory Case Analysis of HRV Indices Across CSS-Classified Segments

**Figure 6:**
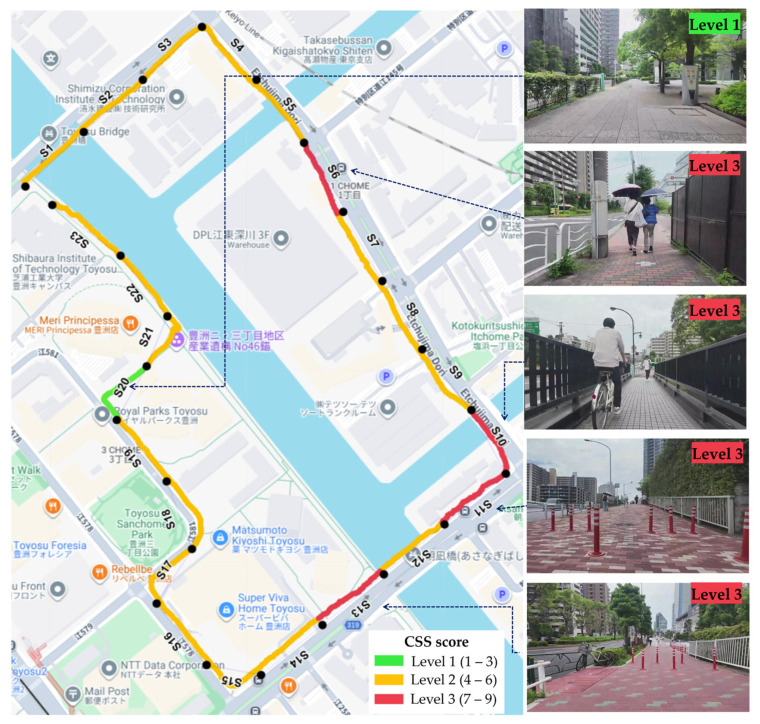
Spatial distribution of stress levels along the experimental route, classified by the CSS. The route is divided into 100 m segments, and colors indicate CSS-based stress levels: Level 1 (1–3, low; green), Level 2 (3–6, moderate; yellow), and Level 3 (6–9, high; red). Black dots indicate the boundaries of the 100 m route segments, and labels (S1–S23) indicate the segment IDs. Representative photographs illustrate typical streetscape conditions corresponding to each stress level.

To illustrate how the proposed CSS-based stress location identification method relates to physiological stress responses, we conducted an exploratory case analysis focusing on representative route segments with clearly contrasting CSS-based stress levels. Route segments were classified into three stress levels (low, moderate, and high) based on the CSS calculated from three stress factors: poor road surface/vibrations, encounters with others, and narrow sidewalks. Figure 6 visualizes the spatial distribution of CSS-based stress levels along the route and provides representative street scenes for low- and high-stress conditions.

Based on the CSS classification, four segments (Segments 6, 10, 11, and 13) were categorized as high-stress segments, whereas Segment 20 was categorized as a low-stress segment. Video inspection revealed common environmental characteristics in the high-stress segments, including brick-paved road surfaces, narrow sidewalks or reduced effective walking space due to poles and other roadside installations, and frequent encounters with pedestrians and cyclists. In contrast, Segment 20 featured a smooth concrete road surface, sufficient sidewalk width, and relatively few encounters with other road users. These observations are consistent with the higher CSS values for Segments 6, 10, 11, and 13 and the lower CSS value for Segment 20. For these reasons, these segments were selected as representative examples for an exploratory case analysis aimed at illustrating a potential correspondence between CSS-based stress classification and HRV changes.

**Figure 7:**
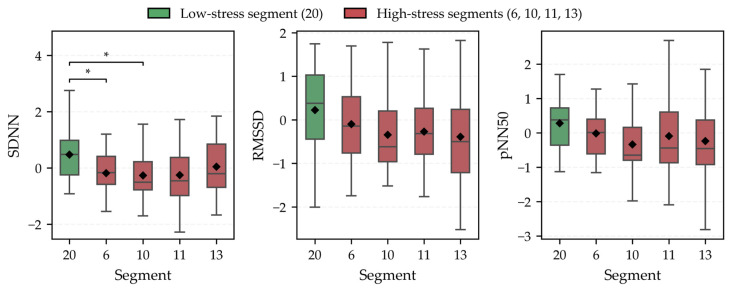
Comparison of z-score–standardized HRV indices (SDNN, RMSSD, and pNN50) between the low-stress segment (Segment 20) and high-stress segments (Segments 6, 10, 11, and 13). Box plots show the median and interquartile range; whiskers indicate the data range, and black diamonds denote mean values. Asterisks (*) denote statistically significant differences (*p* < 0.05).

To examine whether segments classified as high-stress by the CSS tend to exhibit reduced HRV, z-score–standardized HRV indices (SDNN, RMSSD, and pNN50) were compared across the selected segments (Segment 20 vs. Segments 6, 10, 11, and 13) using within-subject analyses (n = 32). Prior to the comparisons, normality was tested for each HRV index within each segment using the Shapiro–Wilk test. Because normality was satisfied for all segments for all three indices (*p* > 0.05), a one-way repeated-measures ANOVA was applied to test whether each HRV index differed across the five segments. The results showed a significant segment effect for SDNN (*p* = 0.0045), whereas no significant differences were observed for RMSSD (*p* = 0.082) or pNN50 (*p* = 0.137). To identify which segments differed for SDNN, post hoc paired t-tests were conducted for SDNN, and *p*-values were Bonferroni-adjusted for multiple pairwise comparisons. As shown in Figure 7, SDNN was significantly lower in Segment 6 (adjusted *p* = 0.0439) and Segment 10 (adjusted *p* = 0.0149) than in Segment 20. Overall, this exploratory case analysis suggests that SDNN may be lower in some CSS-classified high-stress segments (notably Segments 6 and 10) compared with the low-stress segment (Segment 20), providing preliminary support for a potential association between CSS-based stress classification and physiological responses.

## 4. Discussion

### 4.1. Stress Factors During LMD Travel

This study aimed to develop a framework for identifying stress locations during LMD travel by constructing an environment-based stress estimation index using environmental data related to LMD-specific stress factors. Based on post-ride questionnaire responses, three stress factors commonly reported by multiple participants were identified: poor road surface/vibrations, encounters with others, and narrow sidewalks.

Among these factors, poor road surface/vibrations were reported most frequently. This finding is consistent with previous studies showing that vibration significantly affects the comfort of electric wheelchair users [19]. Because LMD users travel in close contact with the road surface and often operate vehicles with relatively small wheels, irregular pavement conditions may directly translate into increased physical discomfort and operational difficulty. These results suggest that road surface conditions may represent an important environmental stress factor during LMD travel.

### 4.2. Evaluation of the Composite Stress Score

This study evaluated the proposed method by examining the association between CSS-based stress estimates and physiological stress responses derived from HRV indices. As noted in Section 2.8, the effective sample size for the segment-level analysis (n = 23) provided approximately 80% statistical power to detect correlations of about |ρ| ≥ 0.56 at α = 0.05. As shown in Table 2, among the 21 CSS–HRV combinations examined, nine showed statistically significant moderate negative correlations. Among these correlations, two exceeded the detectable magnitude suggested by the power analysis: the correlations between CSS (4) and SDNN (ρ = −0.595, 95% CI: −0.83 to −0.22, *p* < 0.01) and between CSS (7) and SDNN (ρ = −0.627, 95% CI: −0.84 to −0.27, *p* < 0.01). Both CSS (4) and CSS (7) represent configurations integrating multiple environmental stress factors. This suggests that CSS values derived from combinations of stress factors may show stronger associations with physiological stress responses than CSS values based on individual factors alone. Both CSS (4) and CSS (7) showed moderate negative correlations with SDNN, indicating that higher CSS values were associated with lower SDNN values. Because lower SDNN reflects increased physiological stress responses, these results suggest that higher CSS values correspond to higher levels of physiological stress.

These findings are consistent with the study hypothesis that an environment-based stress estimation index derived from LMD-specific stress factors is associated with physiological stress responses during travel. Overall, the results suggest the exploratory feasibility of the proposed CSS framework for capturing aspects of physiological stress responses during LMD travel. Furthermore, as shown in Figure 7, comparisons between low-stress and high-stress segments revealed significantly lower SDNN values in some high-stress segments (Segments 6 and 10) compared with the low-stress segment (Segment 20). Because SDNN reflects both sympathetic and parasympathetic nervous system activity, a reduction in SDNN is generally associated with increased stress and autonomic imbalance. These results provide preliminary physiological support for the stress locations identified by the proposed CSS, indicating that some segments classified as high-stress also exhibit elevated physiological stress responses.

### 4.3. Limitations and Future Work

This study has several limitations that should be addressed in future work. First, although three HRV indices (SDNN, RMSSD, and pNN50) were examined, their associations with CSS were not uniform. SDNN showed relatively consistent relationships with vibration-related CSS values, whereas RMSSD and pNN50 did not consistently show significant associations when stress factors were evaluated individually. This difference likely reflects the physiological characteristics of the HRV indices: SDNN represents overall autonomic nervous system activity, whereas RMSSD and pNN50 primarily reflect short-term parasympathetic activity. Because HRV alone was used to validate the proposed CSS-based method, the evaluation of physiological stress responses may be limited. Previous studies have suggested that HRV is more suitable for detecting sustained physiological responses over longer durations, whereas EDA is more sensitive to short-duration and intense stimuli [45]. Therefore, incorporating additional physiological indicators such as EDA together with HRV could provide a more comprehensive assessment of stress responses.

Second, the experiment was conducted in a limited urban environment along a specific route in the Toyosu area of Tokyo. To ensure participant safety and minimize the risk of traffic conflicts, routes involving road crossings were excluded. As a result, more complex or higher-risk travel scenarios that could induce stronger stress responses during LMD travel may not have been included. For example, potentially stressful situations such as proximity to motor vehicles, steep slopes, extreme weather conditions, poorly maintained infrastructure, or areas with different levels of perceived safety were not examined in this study. Although several participants reported experiencing stress or anxiety when passing or encountering pedestrians and cyclists on narrow sidewalks, the range of stress-inducing environmental conditions captured in this study may still be limited. In addition, the stress factors analyzed in this study were derived from participant reports specific to this area, and stressors unique to other urban environments may not have been identified. Consequently, the generalizability of the identified stress factors and the proposed method to different urban contexts remains uncertain. Future studies should therefore collect data across a wider range of urban environments and route configurations to examine whether similar stress patterns are observed and to further validate the robustness and generalizability of the proposed method.

Third, to ensure the safety of the experimental procedures before extending the study to target populations such as older adults or individuals with mobility difficulties, this study recruited healthy adult participants aged 19–32 years. Participants were recruited from a university student population. Most participants had limited or no prior experience using LMDs, which may have influenced their perception of stress during operation. Previous studies have reported age-related differences in stress perception and physiological stress responses [46,47]. Therefore, the findings of this study cannot be directly generalized to elderly or mobility-impaired users of LMDs. Future studies should include actual LMD users, such as elderly participants and individuals with mobility impairments, and employ more diverse and representative samples to examine whether the identified stress factors and the proposed method remain valid across different user groups.

Fourth, stress locations were identified using fixed segment lengths of 100 m. Using shorter segment lengths may enable more precise localization of stress factors and facilitate clearer interpretation of the environmental factors influencing stress. Future work should investigate the effectiveness of the proposed method using different segment lengths.

Fifth, the classification of quantified stress factor levels was determined using a relative approach based on the statistical distribution of the observed dataset. Consequently, the threshold values for “high” and “low” stress are dataset-dependent and may vary if the same procedure is applied in different environments, route configurations, or populations. While this approach enables robust within-dataset comparisons in contexts where absolute stress thresholds are not well established, it may limit direct comparability across studies or locations. Future research should explore the development of standardized or absolute threshold criteria to enhance cross-context generalizability.

Sixth, because the experiments were conducted at different times for each participant, environmental conditions such as weather and pedestrian crowd density may have varied across trials and could influence physiological stress responses. Therefore, HRV indices may have been affected by factors other than the identified stressors. Future research should attempt to standardize environmental conditions as much as possible or collect larger datasets that enable analyses under comparable environmental conditions.

Despite these limitations, this study provides preliminary insights into how environmental factors may contribute to stress during LMD travel and highlights the potential of using environmental data to explore stress locations in urban environments.

## 5. Conclusions

This study explored a framework for estimating potential stress locations during LMD travel using environmental data related to LMD-specific stress factors. Based on post-ride questionnaire responses, three commonly reported stress factors were identified: poor road surface/vibrations, encounters with other road users, and narrow sidewalks. To enable location-specific analysis, the experimental route was divided into 100 m segments. Each stress factor was then quantified using corresponding environmental measures, including vertical acceleration for road surface conditions, the number of encounters with pedestrians and cyclists derived from onboard video recordings, and sidewalk width. Based on these quantified factors, CSS were calculated for each route segment using both individual factors and combinations of multiple factors, and their associations with physiological stress responses were examined using HRV indices. The results indicated that CSS configurations integrating multiple environmental factors tended to show stronger associations with HRV indices than CSS based on individual factors alone. Combinations including poor road surface/vibrations encounters with other road users, and narrow sidewalks showed moderate negative correlations with SDNN, suggesting that higher CSS values may correspond to increased physiological stress responses. Exploratory comparisons between segments with contrasting CSS levels also indicated that some segments classified as high-stress exhibited lower SDNN values than low-stress segments.

Overall, the findings suggest the exploratory feasibility of using environmental data to estimate potential stress locations during LMD travel. However, the generalizability of the findings is limited, as the experiment was conducted along a single urban route and involved only healthy young adult participants. Future studies should examine the proposed framework in more diverse urban environments and with broader user populations, including actual LMD users such as older adults and individuals with mobility impairments, to further evaluate its applicability and robustness.

## Figures and Tables

**Table 1:** Interpretation of the Spearman rank correlation coefficients (ρ) [44].

Spearman Rank Correlation Coefficients (ρ)	Correlation Strength
|ρ| = 1.0	Perfect
0.7 ≤ |ρ| < 1.0	Strong
0.4 ≤ |ρ| < 0.7	Moderate
0.1 ≤ |ρ| < 0.4	Weak
|ρ| = 0.0	None

**Table 2:** Spearman rank correlation coefficients (ρ) with 95% confidence intervals (95% CI) between CSS and HRV indices.

Composite Stress Score (CSS)	RMSSD ρ (95% CI)	SDNN ρ (95% CI)	pNN50 ρ (95% CI)
(1) Poor road surface/vibrations	−0.149 (−0.52, 0.28)	−0.487 (−0.80, −0.05) *	−0.102 (−0.49, 0.34)
(2) Number of encounters with others	−0.385 (−0.68, −0.02)	−0.291 (−0.65, 0.12)	−0.306 (−0.63, 0.04)
(3) Sidewalk width	−0.385 (−0.69, 0.02)	−0.185 (−0.61, 0.28)	−0.385 (−0.67, 0.00)
(4) Poor road surface/vibrations + Number of encounters with others	−0.433 (−0.72, −0.03) *	−0.595 (−0.83, −0.22) **	−0.346 (−0.67, 0.06)
(5) Poor road surface/vibrations + Sidewalk width	−0.306 (−0.63, 0.10)	−0.480 (−0.77, −0.06) *	−0.261 (−0.63, 0.18)
(6) Number of encounters with others + Sidewalk width	−0.508 (−0.78, −0.15) *	−0.345 (−0.63, 0.02)	−0.451 (−0.73, −0.08) *
(7) Poor road surface/vibrations + Number of encounters with others + Sidewalk width	−0.525 (−0.79, −0.12) *	−0.627 (−0.84, −0.27) **	−0.449 (−0.76, −0.03) *

ρ denotes Spearman’s rank correlation coefficient. Values in parentheses indicate 95% confidence intervals estimated using nonparametric bootstrap resampling (5000 iterations). Statistical significance is indicated by * *p* < 0.05 and ** *p* < 0.01.

## Data Availability

The data that support the findings of this study are available on request from the corresponding author due to ethical and privacy restrictions.
